# Student-led research team-building program may help junior faculty increase productivity in competitive biomedical research environment

**DOI:** 10.1186/s12909-020-02396-8

**Published:** 2021-01-04

**Authors:** Marie Bragg, Joshua Arshonsky, Yrvane Pageot, Margaret Eby, Carolyn M. Tucker, Shonna Yin, Emily Goldmann, Melanie Jay

**Affiliations:** 1 Department of Population Health, NYU School of Medicine, 180 Madison Ave, 3-52, New York, NY 10016 USA; 2 Public Health Nutrition Program, NYU School of Global Public Health, New York, NY 10016 USA; 3 Health Psychology Program, University of California–Los Angeles, Los Angeles, CA 90095-1563 USA; 4 Department of Sociology, University of California–Berkeley, Berkeley, CA 94720–1980 USA; 5 Department of Psychology, University of Florida, Gainesville, FL 32603 USA; 6 Department of Pediatrics, New York University Langone Health/Hassenfeld Children’s Hospital, 430 E 34th St., New York, NY 10016 USA; 7 Department of Epidemiology, NYU School of Global Public Health, New York, NY 10003 USA; 8 Department of Medicine, New York University Langone Health, 550 1st Ave, New York, NY 10016 USA; 9 Department of Population Health, New York Harbor Veteran Affairs, 423 East 23rd Street, New York, NY 10010 USA

**Keywords:** Team-building, Junior faculty, Faculty development, Student research assistants, Research teams

## Abstract

**Background:**

Interdisciplinary research teams can increase productivity among academic researchers, yet many junior investigators do not have the training or financial resources to build productive teams. We developed and tested the acceptability and feasibility of three low-cost services to help junior faculty build and maintain their own research teams.

**Methods:**

At an urban academic medical centre, we implemented three types of consultation services: 1) giving talks on evidence-based best practices for building teams; 2) providing easy-to-use team building resources via email; and 3) offering a year-long consultation service—co-led by students—that taught faculty to build and maintain research teams. Our primary outcome was the number of faculty who used each service. For the yearlong consultation service, we asked faculty participants to complete three online self-assessments to rate their leadership confidence, the team’s performance, and which of the consultation components were most helpful. We used descriptive statistics to evaluate faculty assessment scores at three timepoints by comparing median scores and interquartile ranges.

**Results:**

We gave 31 talks on team building to 328 faculty and postdoctoral fellows from 2014 to 2020. Separately, 26 faculty heard about our research team building expertise and requested materials via email. For the consultation service, we helped build or enhance 45 research teams from 2014 to 2020. By the end of the consultation, 100% of the faculty reported they were still maintaining their team. In the initial survey, the majority of participants (95.7%, *n* = 22) reported having no or few experiences in building teams. Further, when asked to rate their team’s performance at 12-months, faculty highly rated many elements of both teamwork and taskwork, specifically their team’s productivity (6/7 points), morale (6/7 points), and motivation (6/7 points). By the end of the program, faculty participants also highly rated two components of the consultation program: recruitment assistance (7/10 points) and provision of team management tools (7/10 points).

**Conclusions:**

For participating faculty, our program provided valued guidance on recruitment assistance and team management tools. The high demand for team-building resources suggests that junior faculty urgently need better training on how to develop and manage their own team.

## Background

In the field of biomedical research, junior faculty (i.e., those with a terminal degree, such as a PhD, MD, DrPH, DO, or any combination of similar degrees) face a challenging grant funding environment [1]. Declining federal funding in the U.S.—coupled with an increasing number of grant application submissions—produces hyper-competition in a research environment where academic medical institutions evaluate junior faculty on their ability to bring in research funding [1, 2]. Although many universities and the National Institutes of Health have implemented programs to address the difficulties in building a research career [3], junior investigators need more support.

Evaluative research on career-building programs for junior faculty suggest that participating faculty feel empowered and gain key research and management skills that they report will help them advance their careers [4]. Few faculty development programs, however, incorporate the value of involving students in faculty research activities. In recent years, studies on the developmental and academic benefits of student research experiences have increased, largely because of the growth of funding support for those experiences. In the US, some institutions provide internship funding for students to participate in research labs or conduct research studies in partnership with faculty [5, 6]. Universities also offer experiential learning courses that provide course credit for participation in a research lab [7, 8]. For students, the gains of this funding are immense. Student research experiences can improve learning (e.g., technical, critical thinking, communication, etc.), retention, degree completion, professional self-confidence, and the likelihood of graduate study [9].

To help facilitate student participation in research, career-building programs should consider incorporating lessons on how to build and sustain a laboratory research team. Students may provide powerful insight in building and maintaining research teams if they have had prior experience as a research assistant in an academic setting. Such students may have unique, first-hand perspectives on what can keep a team motivated and prevent common pitfalls. But no studies have empowered students to play a central role in training faculty members on building and maintaining a research team.

Universities can further support junior faculty by training them to build and manage research teams. Research shows that collaboration increases scientific productivity [10], and teams that include students can increase faculty productivity in publishing articles and receiving grant funding [11]. Yet junior faculty face several barriers to establishing teams, including concerns about the administrative burden of managing a team and a lack of time, money, and team-building training [11].

To help faculty overcome barriers to building teams, we developed and piloted the Research Team Development and Mentorship Training Program (herein referred to as the Program) at New York University School of Medicine (NYUSOM) and New York University (NYU) in August 2014. We implemented three types of consultation services: 1) giving talks on evidence-based best practices for building teams; 2) providing easy-to-use team building resources via email; and 3) offering a year-long consultation service—co-led by students—that taught faculty to build and sustain research teams. The overall objective of the present pilot study was to test the feasibility and acceptability of implementing these three low-cost consultation services.

Our research questions were: 1) Will academic departments and faculty utilize the research team building resources we offer (i.e., is there a demand for these services)?; 2) For academic departments and faculty who utilize the resources, will they become “repeat customers” (e.g., request additional talks annually) or continue to maintain their research team after the conclusion of consultation services?; and 3) Which component(s), if any, of the yearlong consultation service do faculty rate as the most useful team building and management tools?

## Methods

We did not plan to conduct research on building and maintaining research teams. Instead, the demand for these resources appeared and reappeared over time; we developed these consultation services in response to those demands. The program began in 2014 when the lead author (MB) offered to give a research talk on team development in her department as a way to get to know new colleagues from diverse disciplines. After giving that first talk, we did not offer to give it again. Rather, our first type of consultation service—giving talks on evidence-based methods for building and managing a research team—developed in response to invitations to repeat the talk and tailor it to different departments, research centres, and disciplines. These talks were typically an hour long, and involved a PowerPoint presentation and discussion on the following topics: reviewing data on the competitive nature of research environments, particularly in academic medical centres; reviewing research on the benefits of team science; discussing how principles from psychology, teaching, and marketing inform our research team model; providing a list of tools and strategies we use to build and manage our team; and sharing anecdotal evidence on our outcomes and the outcomes of teams we helped (e.g., grant funding successes; publication records; data collection milestones made possible by the team). The primary outcome for this first type of consultation service was quantifying “consumer demand,” which involved calculating the number of talks we gave and the number of people who attended from 2014 to 2020.

The second type of consultation service—sharing easy-to-use team building resources via email—also occurred spontaneously. Each year, junior and senior faculty members, and postdoctoral fellows emailed our team to request any team building and management materials we were willing to share. These individuals had heard about our team model by someone who attended a talk, and had also learned that we freely share the following materials: 1) our guide for recruiting, interviewing, and training students and staff who are diverse in terms of race/ethnicity, sexual orientation, ability status, religion, academic background, research skills, language proficiency, etc.; 2) our “lab manual,” which is a document designed to help staff and research assistants understand our team structure and contribute to our culture of excellence; and 3) hyper-local resources that benefit the investigator (e.g., programs in our university that offer stipends to students for unpaid internships; small grants that are available to students for research). The primary outcome for this second type of consultation service was the number of people who requested these “free” documents and written resources.

The third service involves a consultation program—co-led by students with prior research experience— designed to help junior faculty build their research team while quickly learning how to reduce common administrative burdens. This consultation program offered a “low intensity” and “high intensity” version. In the “low intensity” version, a student consultant from our team would meet with a faculty member who had attended a talk or heard about a talk and wanted more information on team-building. The one-hour meeting would include a “crash course” on how to build a team from start to finish while being mindful of mitigating administrative burdens, including: excessive time spent reviewing resumes, setting up interviews, hosting too many interviews, and spending too much time training new staff or students. We provided timelines and recommended time limits on each task to help junior faculty avoid common time traps.

The “high intensity” version of the consultation service developed in 2017 in response to senior faculty in our department encouraging us to expand our program by applying for funding from the Clinical and Translational Science Institute (CTSI) at our institution. The CTSI awarded $25,000 to our team to examine feasibility and acceptability of the “high intensity” version of the consultation program.

The yearlong “high intensity” program oriented junior faculty to best practices for managing a research team, aided investigators by recruiting, interviewing, and training research assistants, and provided one-on-one coaching to troubleshoot personnel issues. Table 1 overviews the program components and provides expected time commitments.

**Table 1:** Overview of investigator and consultant expectations in the program

Consultant Responsibilities	Investigator Responsibilities
• Orient investigators to team structure model and its benefits • Help investigators design their research teams and recruit research assistants • Interview prospective research assistants and send acceptance and rejection emails to applicants • Help investigators develop lab manuals (document that describes investigators’ expectations, describes research assistants’ responsibilities, and provides resources for writing manuscripts or conducting data analysis) • Troubleshoot management and personal issues that investigators might have	• Attend one-hour needs assessment meeting with student consultant to design research team and learn about management strategies • Complete three, 20-minutre surveys during the program course • Attend at least two interviews with prospective research assistants
**Curriculum Topics Covered During the Program**	
• Organizing and monitoring lab work through activity logs • Providing productive feedback to research assistants, discussing how their performance has progressed and what can be approved • Effectively defining lab expectations (e.g., professionalism, respect, and work ethic) • Writing letters of recommendation • Mentoring research assistants who are considering careers in academic medicine	

We recruited all faculty through announcements at faculty meetings, word-of-mouth, department listservs, and a mentor seminar series hosted by the medical center. Eligibility criteria included junior or senior faculty who were conducting independent research.

During the yearlong program, student consultants dedicated 15 h to each junior faculty member. In exchange, faculty dedicated 5 h to learning team-building skills, interviewing at least 2 research assistants, and completing self-assessments to monitor their progress. The needs assessment described above accounted for 1 of the 5 h. Investigators also spent 1 h leading or observing at least 2, 30-min interviews with research assistants. The third hour was spent completing 3 online self-assessments. The use of the remaining 2 h was at an investigator’s discretion. Most investigators used these additional 2 h to meet with consultants and troubleshoot personnel issues.

The program content stemmed from our collective experiences in building research teams within academic medicine, and from research on teaching pedagogy (e.g., setting clear expectations) [12–14] and research on the role of positive reinforcement in promoting desired behaviours [15, 16]. During the initial needs assessment, consultants provided faculty with a “research staff handbook” that they could then modify and personalize. The handbook content included trainings on research skills such as qualitative coding and conducting literature reviews, as well as written guidelines and policies for team members. Most notably, it instructed faculty on how to set expectations for their team. We introduced them to several variations of research team structures (e.g., having a “lead research assistant” supervise up to four others) [17]. To address the obstacle of limited financial resources, we offered suggestions on how to find students who needed research experience for course credit, how to empower students to apply for small scholarships for unpaid internships, and how to provide a particularly meaningful research experience for volunteer research assistants. Although we emphasize the benefit of paying staff through start-up funding or scholarships whenever possible, we recognize that some faculty do not yet have grants to fund research assistants.

A pre-post design was used to evaluate feasibility, team performance, and confidence in faculty leadership. During both second and third assessment timepoints, faculty also rated the program components they found most helpful. In the needs assessment, before consultants introduced faculty to the team model and overviewed the team-building service, faculty participants were surveyed about their prior team-building training and supervisory experience. At the 6-month and 12-month timepoints, faculty rated their confidence in leadership using a 7-point Likert scale, ranking from 1 for “strongly disagree” to 7 for “strongly agree.” During those same timepoints, faculty also self-reported their satisfaction with teamwork and taskwork. Marks et al. (2001) define taskwork as “what it is that teams are doing” and teamwork as “how they are doing it with each other.” [18] Using this framework, we measured teamwork by asking investigators to rate their team’s goal achievement, project management, productivity, motivation, passion, and communication. We measured teamwork using a 6-point Likert scale, ranking from 1 for “strongly disagree” to 6 “strongly agree.” To measure taskwork, investigators rated their satisfaction with presentations, drafts of IRB applications, drafts of grant proposals, data analysis, drafts of literature reviews, drafts of manuscripts, and other administrative tasks. We measured taskwork using a 7-point Likert scale, ranking from 1 for “strongly disagree” (or “extremely unhelpful) to 7 for “strongly agree.”

We tabulated and exported the assessment data to SPSS to calculate frequencies and medians and to examine trends in team performance and leadership over time. To determine if scores at the 12-month assessment were consistent with those at the 6-month and need assessments, we compared median scores and interquartile ranges. Given our study was designed to test the acceptability and feasibility of the team-building program, we did not conduct analyses to compare the baseline and follow-up assessments. This study was considered exempt by the Institutional Review Board of NYUSOM.

## Results

Our primary outcome—and first research question—focused on quantifying the demand for research team-building resources by recording the number of people who utilized each of the three services between 2014 and 2020. For the first service, which included giving talks on team building, we gave 31 talks to 328 faculty, postdoctoral fellows, staff and students—though the majority in each audience were faculty. For the second service, we provided 26 faculty members with easy-to-use team building resources via email. For the third service, we helped 22 faculty with the “low intensity” consultation program and 23 faculty with the yearlong “high intensity” program that involved meeting with student consultants who helped build the team. Taken together, 399 faculty, postdoctoral fellows, staff, and students expressed interest in learning about research team building from our team.

The second research question asked whether any academic departments, centres, or programs requested an additional talk or follow-up conversation with a subset of faculty. We gave talks to four academic departments; three early career programs within the institution; and two centres (i.e., nine different types of research environments). We were invited to give one or more presentations the following year for all four departments; two of the three early career programs; and one of the centres, meaning we were invited back seven out of nine times.

Across all three services, the most commonly asked questions included: how do I recruit team members without losing too much time for my research activities?; how can I build a team if I don’t have funding to hire personnel?; where do you find student research assistants?; what kind of responsibilities can undergraduate versus graduate/medical students handle?; and do you have any team management materials you could share?

We did not track the number of faculty who developed teams in response to attending a talk or receiving materials via email. For the “low intensity” consultation program, all 22 faculty members successfully built a research team and still maintained that team 1 year later when asked to self-report whether they were still managing their team. For the “high intensity” consultation program, 15 of the faculty were still maintaining their team after 1 year. We could not reach 8 faculty members.

We summarize the results of the feasibility and acceptability of the program below.

In August 2017 we recruited 23 faculty from a variety of departments within NYUSOM and NYU. All 23 faculty participants completed the needs assessment, and 15 completed both the 6-month and 12-month assessments. Table 2 reports the academic backgrounds of faculty members in our sample.

**Table 2:** Needs Assessment and Participant Characteristics (*n* = 23)

	N (%)
Investigators	
*Academic Rank*	
Assistant Professor	16 (69.6)
Associate Professor	2 (8.7)
Clinical Assistant Professor	2 (8.7)
Instructor	1 (4.3)
Clinical Associate Professor	2 (8.7)
*Academic Discipline*	
Public Health	12 (52.2)
Obstetrics and Gynaecology	1 (4.3)
Paediatrics	2 (8.7)
Internal Medicine	2 (8.7)
Gastroenterology	3 (13.0)
Neurology	2 (8.7)
Orthopaedic Surgery	1 (4.3)
*Any prior team-building and mentor training*	
Yes	0 (0.0)
*Any prior supervisory experience*	
Yes	22 (95.7)
*Preferred team size*	
1–2 research assistants	13 (56.6)
3–4 research assistants	5 (21.7)
5 or more research assistants	5 (21.7)
*Preferred type of research assistants*	
Undergraduate students	22 (95.7)
Graduate students	21 (91.3)
Medical students	11 (47.8)
Part-time staff	12 (52.2)
Full-time staff	5 (21.7)
*Special research needs (*e.g.*, language proficient, etc)*	
Language proficiency	5 (21.7)
Data analysis	1 (4.3)

### Feasibility

No faculty participants had prior team-building training, but almost all (95.7%) had prior supervisory experience. In the needs assessment, more than half of the faculty participants (56.5%) reported wanting a team of 1–2 research assistants. Five participants (21.7%) wanted 3–4 research assistants, and 5 (21.7%) wanted 5–6 research assistants. Most investigators reported wanting teams of undergraduate students (95.7%) and/or graduate students (91.3%). At 6 months, 6 investigators reported having teams of 1–2 research assistants, and 6 had 3–4 research assistants. Three investigators reported having 5 or more research assistants. The majority of investigators had teams composed of undergraduate students (80.0%; *n* = 12). At 12 months, investigators reported similar team sizes. Six investigators had teams of 3–4 research assistants, and 5 had teams of 1–2 to research assistants. Most investigators reported having teams composed of undergraduate students (60.0%; *n* = 9) and/or graduate students (53.3%; *n* = 5).

### Team performance

In the 6-month and 12-month assessments, investigators highly rated several measures of team performance with increased ratings noted for some measures of teamwork and taskwork. As shown in Table 3, investigators reported increased agreement with their team’s productivity and efficiency (median score increase, 5.0 to 6.0; Interquartile Range [IQR] = 1.0[6.0–5.0] and 1.0[6.0–5.0], respectively), team motivation and enthusiasm (median score increase, 5.0 to 6.0; IQR = 1.0[6.0–5.0] and 1.0[6.0–5.0], respectively), and their team’s belief that they are contributing to valuable research (median score increase, 5.0 to 6.0; IQR = 1.0[6.0–5.0] and 1.0[6.0–5.0], respectively). Investigators’ satisfaction with their teams’ drafts of literature reviews (median score increase, 5.0 to 6.0, IQR = 1.0[6.0–5.0] and 2.0[7.0–5.0], respectively) and drafts of manuscripts (median score increase, 4.0 to 5.0, IQR = 2.0[6.0–4.0] and 2.0[6.0–4.0], respectively) also increased from 6 month into the Program to 12 months. There were a couple of team outcome measures for which median score decreased. Investigators’ satisfaction with their teams’ drafts of IRB applications (median score decrease, 6.0 to 5.5, IQR = 2.0[6.0–4.0] and 2[6.5–4.5], respectively) and drafts of grant proposals (median score decrease, 4.5 to 4.0, IQR = 2[5.5–3.5 and IQR = 4[7.0–3.0], respectively]) decreased between the 6-month and 12-month assessments.

**Table 3:** Self-Reported Ratings of Team Performance By Investigators at 6-Months and 12-Months (*n* = 15)

Team Performance	6-Months					12-Months				
	Mean	SD	Q1	Median	Q3	Mean	SD	Q1	Median	Q3
**Teamwork**^a^										
Since developing my research team, interns have met or exceeded the goals we have established in our project timelines (e.g., IRB applications, grant submissions, participant recruitment, etc.)	5.0	0.9	5.0	5.0	6.0	5.3	0.8	5.0	5.0	6.0
My research team has established an effective method for managing ongoing project tasks and abides by it (e.g., activity log, weekly check-in’s, etc.)	5.2	1.1	5.0	5.0	6.0	4.9	1.2	4.0	5.0	6.0
Overall, my research team is productive and efficient in carrying out their assigned tasks and submits tasks/projects by the assigned due date.	4.8	0.8	5.0	5.0	6.0	5.4	0.7	5.0	6.0	6.0
My research team members are motivated and enthusiastic in completing their tasks and projects.	5.3	0.6	5.0	5.0	6.0	5.7	0.5	5.0	6.0	6.0
My research team members believe they are contributing to valuable research and are passionate about their work.	5.3	0.5	5.0	5.0	6.0	5.3	0.9	5.0	6.0	6.0
My research team has established an effective method of managing communication among the team.	4.9	1.0	4.0	5.0	6.0	4.9	1.3	5.0	5.0	6.0
Weekly lab meetings are organized, productive, and promote participation from team members (e.g., journal club, discussing relevant current events, student led trainings for data collection methods, etc.)	4.5	0.7	4.0	5.0	5.0	4.9	0.9	4.0	5.0	6.0
The research assistants in my lab have helped to make me more productive in my research (e.g., paper submissions, presentations, data collection, etc.)	4.5	1.1	4.0	5.0	6.0	5.2	0.8	5.0	5.0	6.0
**Taskwork**^a^										
Team Productivity	5.4	1.0	5.0	6.0	6.0	5.6	1.6	5.0	6.0	7.0
Morale	5.8	0.7	5.0	6.0	6.0	5.8	1.4	5.0	6.0	7.0
Presentations	4.9	1.1	4.0	5.0	6.0	5.1	1.4	5.0	5.0	6.0
Drafts of IRB applications	5.3	1.5	4.0	6.0	6.0	4.9	2.0	4.5	5.5	6.5
Drafts of grant proposals	4.5	1.3	3.5	4.5	5.5	4.0	1.9	3.0	4.0	7.0
Data analysis	5.2	0.8	5.0	5.0	6.0	4.8	2.0	3.5	5.0	7.0
Drafts of literature reviews	5.2	1.1	5.0	5.0	6.0	5.5	1.5	5.0	6.0	7.0
Drafts of manuscripts	4.2	1.8	4.0	4.0	6.0	4.6	1.4	4.0	5.0	6.0
Other administrative tasks	5.4	1.4	5.0	6.0	6.0	5.5	1.2	5.0	6.0	6.0

^a^Scale: 1, Strongly disagree; 4, neither; 7, strongly agree

### Confidence in leadership

Table 4 also lists faculty’s self-reported ratings of their leadership. Median scores for all leadership skills either increased or stayed constant over the course of the Program. Investigator’s self-assessment of their ability to effectively and politely terminate contracts with research assistants (median score increase, 4.0 (IQR = 2.0[4.0–2.0]) to 5.0 (IQR = 3.0[7.0–4.0]) to 6.0 (IQR = 3.0[6.0–3.0])) and ability to delegate tasks (median score increase, 4.0 (IQR = 1.0[5.0–4.0]) to 5.0 (IQR = 2.0[5.0–3.0]) to 6.0 (IQR = 2.0[7.0–5.0])) showed the greatest increases in median score during the program.

**Table 4:** 10-Item Self-Assessment of Investigators’ Leadership Skills at Needs Assessment, 6-Months, and 12-Months

Questions^a^	Needs Assessment (n = 23)					6-Months (n = 15)					12-Months (n = 15)				
	Mean	SD	Q1	Median	Q3	Mean	SD	Q1	Median	Q3	Mean	SD	Q1	Median	Q3
1. I believe I have been successful with effectively managing emails, meetings, delegating tasks, and handling other administrative needs.	4.0	1.7	2.0	4.0	5.0	4.0	1.3	3.0	4.0	5.0	4.9	1.1	4.0	5.0	6.0
2. I believe I have been successful with problem-solving common challenges that can arise when supervising a research team (e.g., tardiness, poor quality work, unexcused absences, etc.)	4.0	1.6	3.0	4.0	5.0	4.9	1.0	4.0	5.0	5.0	4.8	1.6	3.0	5.0	6.0
3. I believe I have been successful in effectively and politely terminating a contract with an intern or staff member who has repeatedly had poor performances.	3.4	1.2	2.0	4.0	4.0	5.0	1.6	4.0	5.0	7.0	4.8	2.0	3.0	6.0	6.0
4. I believe I have been successful with inspiring my research team in a way that maintains morale and helps achieve progress on various research studies.	4.6	1.6	3.0	5.0	6.0	5.1	1.1	4.0	5.0	6.0	5.5	1.3	5.0	6.0	6.0
5. I believe I have been successful with generating ongoing, meaningful tasks for interns and staff.	4.2	1.4	3.0	4.0	5.0	4.7	1.2	4.0	5.0	5.0	5.5	1.4	5.0	5.0	7.0
6. I believe I have been successful in knowing what kinds of tasks can be delegated to an undergraduate students, graduate student, or experienced staff member.	4.2	1.3	4.0	4.0	5.0	4.6	1.6	4.0	5.0	6.0	5.1	1.7	3.0	6.0	6.0
7. I believe I have been successful in providing meaningful professional development mentoring (e.g., guiding mentees on getting into graduate school, building their resume, etc.)	5.2	1.1	5.0	5.0	6.0	5.5	0.9	5.0	5.0	6.0	5.5	1.1	5.0	6.0	6.0
8. I believe I have been successful in providing written feedback on submitted documents without spending too much time on those documents.	4.6	1.3	4.0	5.0	5.0	4.6	1.2	4.0	5.0	5.0	4.3	1.4	4.0	5.0	5.0
9. I believe I have been successful in implementing the most efficient ways to train research assistants on basic tasks (e.g., literature searches, developing IRB applications, entering data, etc.)	3.3	1.4	2.0	3.5	4.0	4.7	1.6	4.0	5.0	6.0	4.8	1.4	4.0	5.0	6.0
10. I believe I have been successful in designing and lead meaningful, interactive team meetings (i.e. making meetings engaging and useful for team)	3.9	1.5	3.0	4.0	5.0	4.6	1.2	3.0	5.0	5.0	4.8	1.7	4.0	5.0	6.0

^a^Scale: 1, I disagree; 4, I’m doing okay in this area; 7, I agree

### Faculty feedback on the program

The third research question asked which component(s), if any, of the yearlong consultation service do faculty rate as the most useful team building and management tools? Table 5 shows investigators ratings of the most helpful components in the program. At the conclusion of the program, the most highly ranked components were the overview of Google Drive (median = 7.0, IQR = 2.0[7.0–5.0]), the overview of the research positions (e.g., research assistant and lead research assistant) (median = 7.0, IQR = 1.5[7.0–5.5]), recruitment assistance (median score = 7.0, IQR = 0[7.0–7.0]), and the provision of sample documents like research assistant applications and project deliverable worksheets (median = 7.0, IQR = 1.0[7.0–6.0]). Although some investigators reported having some difficulty troubleshooting the performance issues of some research assistants, the majority of investigators reported that the consultation service was a key factor in reducing the administrative burden of building a team.

**Table 5:** Helpfulness Ratings of Program Components (n = 15)

Items^a^	6-Months			12-Months		
	Q1	Median	Q3	Q1	Median	Q3
Lab Manual	5.0	6.0	7.0	5.0	6.0	7.0
Overview of Google Drive	4.0	6.0	6.0	5.0	7.0	7.0
Overview of positions (e.g., intern, lead intern)	5.0	5.5	7.0	5.5	7.0	7.0
Overview of team meetings	5.0	5.5	6.0	5.0	6.5	7.0
Recruitment	6.0	7.0	7.0	7.0	7.0	7.0
Intern orientation	5.0	6.0	7.0	6.0	6.5	7.0
Sample documents (e.g., research assistant application template, project deliverable worksheets, etc.)	5.0	6.5	7.0	6.0	7.0	7.0
Tips to enhance team productivity/manage research assistants efficiently	4.5	5.0	6.0	5.0	6.5	7.0

^a^Scale: 1, extremely unhelpful; 4, neither; 7, extremely helpful

## Discussion

To our knowledge, the Research Team and Mentorship Development Program is the first to incorporate a consultation service led by experienced student researchers. We demonstrate that nearly 400 faculty of various rank expressed interest in the program, and even more wanted to share our program with others in their academic departments or centres. We had more than 12 additional talks in academic development programs or centres within the medical research institution. We also show that our unique consultation service to help faculty build research teams was widely received by many faculty participants. Many of them rated the recruitment of research assistants and team-building model as the most helpful components of the consultation service. These results support findings of previous studies evaluating career development programs for junior faculty and align with faculty’s perceived barriers and limitations to building a student research team [19–23].

The student-led component of our consultation team-building program could be led by experienced senior faculty. But that approach does not substantially reduce the administrative burdens of team-building, and research on mentoring shows that having students on a research team can make it easier to perform projects [24]. And when graduate students or more senior undergraduate research assistants take leadership roles within teams, this provides a built-in hierarchy in which students with more experience can guide students with less experience [24]. All the while the junior investigator can supervise and tackle the research tasks that only they can handle without having to guide the less experienced research assistants through tasks like literature reviews or data entry, therefore potentially increasing their productivity. If funding were not available at the institutional level, then one option could be for departments to use discretionary funding to provide group workshops for interested faculty. And, if there were no funding at both the institutional or departmental level, “expert team leaders” (i.e., junior or senior faculty who have excelled in leading a research team) could provide a few workshops per year during any existing departmental grant-writing groups, peer mentoring networks, or other junior faculty support systems.

Our feasibility pilot study has several limitations. For 1 year we piloted our team-building program at a large academic medical center in a major American city, meaning there are limits to generalizability. Most faculty had no prior team-building training, so it might be difficult for junior and more senior faculty to advance their research teams and collaborate across disciplines. Our study team did not include measures of the multiculturalism in our surveys, meaning we did not quantify this key feature of research teams. Multicultural approaches to research facilitate student learning and invites the inclusion of diverse social and cultural perspectives [25]. Quantifying the level of diversity in research teams, therefore, is essential for better understanding how this consultation service can increase multicultural approaches to building teams and conducting culturally-sensitive research. We also did not measure the degree of previous supervision or mentorship among investigators. Given 95.7% of investigators had prior supervisory experience, it is possible that this provided an advantage in their ability to build and maintain their team as part of this consultation service. Future studies should ask more granular questions regarding how many years of supervision and mentorship the faculty had, as well as the types of personnel they supervised or mentored (e.g., undergraduate students; full-time staff members). Finally, objective tools to measure improvements among faculty were not used in this feasibility pilot. It is possible that such measures would show vastly different results, both positive or negative. To comprehensively examine the impact of a student led team-building program on junior faculty, future studies should incorporate objective measurement tools such as the Collaborative Productivity Scale or Cross-Disciplinary Collaboration Activities Scale [26].

Despite having 23 investigators enrol and participate in the yearlong consultation, only 15 participating faculty completed both the 6-month and 12-month assessments. This moderate to low retention is consistent with findings from previous team-building studies. In career development programs spanning a year or more, participating faculty often drift away by first ceasing to show up and by then by stopping to respond altogether. Other times, faculty become overwhelmed and overworked and dropout, overestimating the commitment it takes to maintain a research team. And while it does take a concerted effort and active approach to dedicate your time to building research teams, it, in the end, can be systematized in a way so that it becomes second nature. The troves of students that enter our research teams signal the promise of our consultation service in strengthening the pipeline of future researchers and clinicians in academic medicine.

Implementing similar team-building programs at other academic medical institutions could help support more junior faculty face the hypercompetitive research environment. Because not all institutions have the capacity to launch a program like ours, we will develop a publicly available, online training tool. This online tool could introduce our research team model and consultation service and provide examples of management tools—such as team manuals, project deliverables worksheets, and research assistant applications. The online tool will also include a feature allowing its visitors to provide feedback.

## Conclusions

Our program offers juniors an experience to build research teams through three types of low-cost services. For the year-long consultation service, experienced students functioned as study consultants and introduced faculty to an adaptable research model, provided them with team management tools, and helped faculty recruit research assistants. Such components help address some of the reported obstacles to building a team. Through these program features and others, the authors hope to help more junior faculty face the challenges of academic research and to provide more meaningful research experiences for students.

## Data Availability

For this study the data analysed are available from the corresponding author on reasonable request.

## Abbreviations

PhD: Doctor of Philosophy
MD: Doctor of Medicine
DrPH: Doctor of Public Health
DO: Doctor of Osteopathic Medicine
NYUSOM: New York University School of Medicine
NYU: New York University
CTSI: Clinical and Translational Science Institute
IQR: Interquartile Range
